# Molecular evidence of *Anaplasma phagocytophilum* in wild boar (*Sus scrofa*) in Belgium

**DOI:** 10.1186/1746-6148-10-80

**Published:** 2014-04-02

**Authors:** Adrien Nahayo, Marjorie Bardiau, Rosario Volpe, Jessica Pirson, Julien Paternostre, Thomas Fett, Annick Linden

**Affiliations:** 1 Surveillance Network of Wildlife Diseases, Department of Infectious Diseases, Faculty of Veterinary Medicine, University of Liege, Liege, Belgium; 2 Bacteriology, Department of Infectious Diseases, Faculty of Veterinary Medicine, University of Liege, Liege, Belgium

**Keywords:** Wild boars, *Ixodes*, *Anaplasma phagocytophilum*, Belgium

## Abstract

**Background:**

*Anaplasma phagocytophilum* is a tick-borne pathogen of veterinary and human importance. Both ticks as vectors and vertebrates as reservoir hosts are essential for the cycle maintenance of this bacterium. Currently, the whole range of animal species reservoirs for *A. phagocytophilum* in natural environment is still unknown. Therefore, the aim of this study was to estimate the prevalence of infection with *A. phagocytophilum* in the wild boar population in southern Belgium.

**Results:**

In the frame of a targeted surveillance program, 513 wild boars were sampled during the hunting season 2011. A nested 16S rRNA PCR was used to screen the presence of *A. phagocytophilum* DNA in spleen of boars. Within 513 samples, 5 (0,97%) were tested PCR positive and identification was confirmed by sequencing.

**Conclusions:**

This study gives the first insight of presence of *A. phagocytophilum* in wild boars in southern Belgium.

## Background

*Anaplasma phagocytophilum* is a tick-transmitted pathogen causing granulocytic anaplasmosis in human (HGA), equine and canine granulocytic anaplasmosis and tick-borne fever in ruminants [1]. The epidemiology of *A. phagocytophilum* is closely related to the ecology of its main vector, namely the hard tick of the *Ixodes* genus. The most representative vectors include *I. ricinus* in Europe, *I. persulcatus* in Asia, *I. scapularis* in Eastern and Mid-Western United States and *I. pacificus* in California [2]. Although their vectorial implication has not been evidenced, *A. phagocytophilum* has also been detected in non-*Ixodes* hard ticks, such as *Boophilus, Dermacentor, Hyalomma*, and *Rhipicephalus*[3-5]. The bacterium is transstadially transmitted by the vector ticks but there is no evidence of transovarial transmission [6,7]. Therefore, both mammalian reservoirs and vector ticks are essential for the cycle maintenance of *A. phagocytophilum*.

In Europe, wild cervids in particular roe deer (*Capreolus capreolus*) are recognized as competent hosts for *A. phagocytophilum*[8,9]. The presence of this bacterium has also been reported in other wild animals, such as birds and rodents [3,6,8] but the whole range of animal species reservoirs for this bacterium in natural environment is still unknown. The presence of *A. phagocytophilum* in wild boars has been documented in several Eastern European countries [10-13], and also in Japan [14]*.* However, the role of wild boars in the epidemiology of *A. phagocytophilum* is not fully elucidated.

In Belgium as in other European countries, a steady increase of wild boar populations has been observed over the last twenty years. From 1987 to 2007, according to the official census of the Department of Nature and Forestry, the estimated hunting bags of wild boar in southern Belgium (16,844 km^2^) increased from 6,000 to 22,000 heads [15]. The higher abundance of wildlife and the increased contact between wildlife, domestic animals and human populations raises the risk for outbreaks of tick borne diseases, and then the difficulty of implementing surveillance and control measures [16].

The granulocytic anaplasmosis due to *A. phagocytophilum* is the most widespread tick borne infection in animals [17]. Although the disease is known quite a long time in veterinary medicine, the observed increasing number of clinical and/or asymptomatic cases of HGA during the two last decades mainly in United States [18] but also in Europe, has generated growing public health interest in this zoonosis. Recently, Cochez and co-workers reported 111 confirmed cases of HGA between 2000 and 2009 in Belgium [19]. Despite interest for this pathogen, epidemiological data are scarse and there is few informations concerning *A. phagocytophilum* infections in wild boars in Western Europe, even though they are one of the most abundant big game species.

Therefore, the aim of this study was to estimate the prevalence of infection with *A. phagocytophilum* in the wild boar population in southern Belgium using PCR targeting gene coding for the 16S ribosomal RNA.

## Results and discussion

Within 513 spleens tested, 5 samples were tested positive and confirmed to be infected with *A. phagocytophilum* corresponding of CE18 (Genbank accession number GQ450278.1), and Dama 35 (Genbank accession number GQ450276.1) strains by sequencing analysis. This prevalence of 0.97% (95% IC: 0.12 - 1.82) in wild boars is surprisingly low compared to that previously detected in roe deer (85.6%) collected in the same region [20]. This discrepancy is also reported in other European studies. Wild cervids are important hosts for *I. ricinus*, the main vector of *A. phagocytophilum.* These species, especially roe deer, are largely infected by this bacterium [9,10,21]. In certain regions of Germany, the prevalence of *A. phagocytophilum* in roe deer reached 95% [22] and even 100% [23]. The role of these ungulates as competent reservoir for *A. phagocytophilum* is established [9,11].

Regarding wild boars, the situation is less clear. As in the present study, *A. phagocytophilum* has been reported with low prevalence in other countries such as the Czech Republic [11], Slovenia [12] and Romania [24] with rates of infection of 3 to 4%. Higher prevalences were detected in wild boars sampled in Poland (12%) and in the Czech Republic (14%) in region close to the Austrian border [13,10]. In contrast, studies performed in Austria and North of Spain failed to detect any infected boars by *A. phagocytophilum*[21,25]. This low prevalence in wild boars supports the hypothesis that these animals can be naturally infected but capable to control infection. One of the pathways for controlling *A. phagocytophilum* infection may be the activation of innate immune responses and cytoskeleton rearrangement in order to promote phagocytosis and autophagy [26].

*A. phagocytophilum* is a single species but there is a genetic heterogeneity within the species and many closely related strains are described that differ in vectors, host preferences, geographical distribution but also pathogenicity [27]. All strains circulating in wild hosts are not infectious for humans. As an example, the main reservoir for human pathogenic strains in North America is the white-footed mouse (*Peromyscus leucopus*) while the white-tailed deer (*Odocoileus virginianus*) is reservoir for strains non infective for humans [28]. In Europe, cervids harbor mostly *A. phagocytophilum* strains that are not detected in humans [10,21] but one study [22] reported that red deer may be infected with strains infectious for humans. Interestingly, human pathogenic strains of *A. phagocytophilum* have been detected in wild boars in Slovenia, Poland and the Czech Republic, suggesting that these ungulates may represent a potential reservoir for strains associated with HGA [10,12,13]. Further studies are needed to elucidate the role of wild boars in the epidemiology of *A. phagocytophilum* and to determine if strains detected in wild boars in Belgium are human-pathogenic strains.

## Conclusions

Our data show that wild boars can be naturally infected by *A. phagocytophilum* in southern Belgium. The low prevalence infection detected in the present work is in accordance with results from previous studies and suggest that wild boars could play a less important role than cervids as reservoir for *A. phagocytophilum.* However, as they are particularly abundant, if they were found to harbor strains pathogenic to humans, they may represent a significant source of infection for people carrying out outdoor activities.

## Methods

### Study area

**Figure 1:**
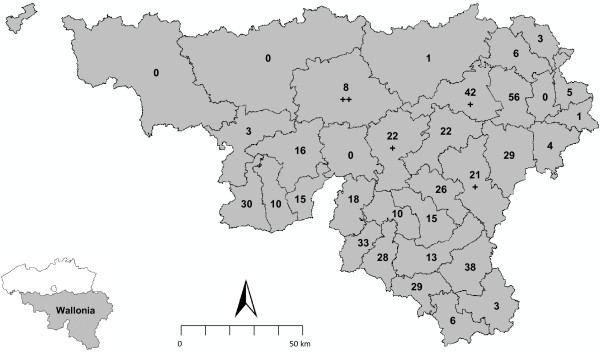
**Geographic distribution of wild boars sampled in southern Belgium (Wallonia) in 2011.** The map shows the number of boars per forest district and “+” indicates the 5 *A. phagocytophilum* PCR positive animals.

The study was conducted in Southern Belgium (Region of Wallonia, N44°42′, E06°37′ to N49°42′, E06°37′). Animals were sampled in 29 forest districts (13,000 Km^2^) known to shelter wild boars (Figure 1).

### Target population and sampling design

In region of Wallonia, the population of wild boars was estimated to 25,000 heads in 2011 [15]. A two-stage cluster sampling was realised. Firstly, some hunting areas were randomly chosen in each forest district and, secondly, some animals were randomly sampled in each hunting area. Forest districts are different related to their wild boars density but an ideal proportional allocation was not possible due to field constraints.

In the frame of a targeted surveillance program previously described [29], 513 boars were sampled during the hunting season (from 1st October to 31st December) in 2011. They had been shot by accredited hunters in the framework of the *Wallonia Surveillance Network of Wildlife Health* that had been established a decade ago by a specific ministerial decree (n° 43.01.03/DNE/2010 from the Public Service of Wallonia). Hunters, scientists and the public authorities concerned all comply with the Convention on Biological Diversity and the Convention on the Trade and Endangered Species of Wild Fauna and Flora.

All animals were necropsied in the field, within 2 to 3 hours after shot. Individual postmortem examinations included determination of sex, age, body weight, and body condition. Age was determined on the basis of tooth eruption patterns and weight [30]. Animals were classified as juveniles (less than 1 year old), sub-adults (between 12 and 24 months old) and adults (over 2 years old) (Table 1). After examination of the intact whole body, the abdominal, thoracic, and naso-buccal cavities and corresponding organs were checked. Afterwards, a piece of spleen tissue was collected and transported to the laboratory within 24 hrs for storage at −20°C until DNA extraction.

**Table 1:** Distribution of hunter-killed wild boars (n =513) sampled in southern Belgium in 2011 (316 in October, 185 in November and 12 in December)

**Age group**		**Gender**			**Total**
	**Females**	**Males**	**Gender nc.**		
**Adults: > 2 years**	73(1)	76	1	**150**
**Sub adults: 1–2 years**	32	34	0	**66**
**Piglets: > 6 months**	87(2)	97(1)	3	**187**
** < 6 months**	34(1)	22	3	**59**
**Age group nc.**	24	25	2	**51**
**Total**	**250**	**254**	**9**	**513**

In parentheses: 5 Wild boars tested PCR positive for *A. phagocytophilum* (2 animals in October and 3 in November 2011).

nc.: not communicated.

### DNA extraction and PCR amplification

From 50 mg of spleen pulp tissue, total DNA was extracted using the DNAzol® reagent purification method (Invitrogen™) according to the manufacturer’s protocol. After extraction, quality and quantity of yielded DNA were evaluated using a spectrophotometer (NanoDrop ND-1000, Thermo Scientific) and then stored at −20°C until PCR reaction.

The yield DNA was tested with a 16S rRNA nested PCR. The first PCR amplifies a 1462 bp fragment common to all *Anaplasma* and *Ehrlichia* species using the specific primers EC9 (5′-TACCTTGTTACGACTT-3′) and EC12A (5′-TGATCCTGGCTCAGAACGAACG-3′) while the second using the specific primers SSAP2F (5′-GCTGAATGTGGGGATAATTTAT-3′) and SSAP2R (5′-ATGGCTGCTTCCTTTCGGTTA-3′) amplifies a specific 641 bp fragment of the 16S rRNA *Anaplasma phagocytophilum* gene [31]. The PCR protocol has been adapted from the original one. The first amplification was performed as following: preliminary denaturation of 2 min at 95°C; 40 cycles, each cycle consisting of: 30s at 95°C, 30s at 52°C, and 90s at 68°C; final elongation of 5 min at 68°C. The second amplification was performed as following: preliminary denaturation of 2 min at 95°C; 40 cycles, each consisting of: 1 min at 95°C, 1 min at 56°C, and 1 min at 68°C; final elongation of 5 min at 68°C. One μl of DNA template in first PCR and one μl of first amplification products in second were screened, using the above mentioned primers and 0.25 μl of Taq DNA polymerase (NEB), following the manufacturer’s instruction. Negative and positive controls were performed by adding either doubly distilled water or DNA from cultured *Anaplasma phagocytophilum* diluted at 1/100 instead of DNA samples. The amplified products were preliminarily electrophorezed on E-gel (Invitrogen), and visualized after agarose gel electrophoresis in standard conditions, stained with 2% Midori green DNA stain, (NIPPON Genetics, GmbH) and then visualized under UV light.

The positive amplified products were purified and sequenced by GATC Biotech.

## Competing interests

The authors declare that they have no competing interests

## Authors’ contributions

AN performed the field work, collected the samples, analysed the data and drafted the manuscript. MB and TF carried out and supervised the laboratory work including molecular analysis and sequencing. RV, JP and JP collaborated for the field sampling. AL was the project leader, supervised the study and contributed to the draft. All authors read and approved the final manuscript.
